# Cognitive and Linguistic Development in Children and Adolescents

**DOI:** 10.3390/children12010012

**Published:** 2024-12-25

**Authors:** Irene Cadime, Iolanda Ribeiro, Maria Luisa Lorusso

**Affiliations:** 1 Research Centre on Child Studies, University of Minho, 4710-057 Braga, Portugal; 2 School of Psychology, University of Minho, 4710-057 Braga, Portugal; iolanda@psi.uminho.pt; 3 Unit of Child Psychopathology, Scientific Institute IRCSS E. Medea, 23842 Bosisio Parini, Italy; marialuisa.lorusso@lanostrafamiglia.it

## 1. Introduction

Cognitive development encompasses the mental processes involved in acquiring, organizing, and utilizing knowledge [1]. A substantial body of research has provided evidence that a range of factors influence cognitive growth, including individual and environmental influences [2,3]. Moreover, particular attention has been paid to the intricate relationship between cognitive and linguistic development. Research consistently highlights the presence of deficits in cognitive skills—such as memory, executive functions, reasoning, and problem solving—among children with linguistic impairments or reading and writing disorders [4,5,6,7]. This Special Issue aims to present recent contributions that enhance our understanding of both typical and atypical cognitive and linguistic development, with a focus on the interplay between these domains. It includes nine research articles presenting studies involving children and adolescents at various developmental stages from various populations (reflecting different countries, languages, cultural backgrounds, and clinical/non-clinical samples), including longitudinal research that tracks outcomes over time. In the next section, we briefly summarize each of the nine contributions.

## 2. An Overview of Published Articles

The study by Trinczer et al. (contribution 1) explores the contributions of sustained attention and response inhibition to reading comprehension among Japanese adolescents via a sample of 73 ninth-grade students. Consistently with recent theoretical models of reading, such as the Active View of Reading [8], the results revealed that both sustained attention and response inhibition significantly predicted reading comprehension, emphasizing their importance in non-alphabetic languages like Japanese, which involve logographic and syllabic scripts. Gender differences were observed in response inhibition, with males making more commission errors. These findings highlight the universal role of attention in reading across diverse orthographic systems and suggest that response inhibition is particularly relevant for complex writing systems. These insights support the potential for targeted cognitive training to enhance reading skills.

The study by Kuhn et al. (contribution 2) compared three executive function (EF) batteries—EF Touch, NIH Toolbox Early-Childhood Cognition Battery (NT-ECCB), and Wechsler Preschool and Primary Scale of Intelligence IV (WPPSI-IV)—in a diverse sample of 3- to 5-year-old children in the USA. The results showed that the batteries performed similarly, with moderate correlations between them. However, EF Touch exhibited some ceiling effects in 5-year-olds, making it particularly suitable for younger children, whereas NT-ECCB showed increased reliability for older preschoolers. Demographic differences, such as Hispanic children scoring lower across all batteries, highlight the need for more inclusive EF assessments. Overall, the findings of this study underscore the importance of balancing cost, accessibility, and population-specific suitability when selecting an EF battery.

The study by Mousoulidou and Paterson (contribution 3) examines how children (ages 6–8) and adults interpret ambiguities in numerically quantified expressions, focusing on whether references are linked to previous entities (subset reading) or introduce new ones (new-set reading). Across six experiments involving 249 children and 50 adults, differences in interpretation were observed: adults predominantly favored the subset reading, reflecting an integration of text elements, while children showed a strong preference for the new-set reading, even in contexts explicitly designed to cue subset interpretation. The children’s responses suggested challenges regarding text integration and comprehension rather than outright inability. In summary, the findings of this study emphasize significant developmental differences in cognitive and linguistic processing, with implications for understanding language comprehension strategies in children.

The study by Cano-Villagrasa et al. (contribution 4) examines executive functioning and language skills in 150 children with autism spectrum disorder (ASD), epilepsy, and both (ASD + epilepsy). The findings reveal that children with both conditions exhibit more severe impairments in executive functions (e.g., planning, working memory, inhibition, etc.) and language (e.g., semantics, morphosyntax, pragmatics, etc.) than children with either condition alone. Epilepsy alone was associated with relatively better cognitive and linguistic outcomes than ASD or the combined condition. Thus, the findings support the idea that ASD has a detrimental effect on executive skills, while epilepsy has an additive negative effect on language and executive functions in children with ASD, underscoring the need for tailored educational strategies to address deficits in social communication and executive skills in this population.

The study by Andres et al. (contribution 5) investigates the genetic basis of specific language impairment (SLI) using whole-exome sequencing and family-based analyses. This study involved 74 individuals from eight families and identified genetic variants in several novel genes as well as genes previously associated with language abilities. The findings emphasize the heritability of grammar-specific deficits, with the identified variants playing potential roles in neural development and ribosomal functions. This study highlights that grammar impairments may have specific genetic underpinnings and that multiple genetic variants, rather than a single causal gene, contribute to SLI. The authors advocate for expanded genetic and phenotypic analyses to refine our understanding of the molecular pathways influencing language acquisition.

The study by Cruz et al. (contribution 6) describes the development and investigation of the psychometric properties of a screening tool designed to assess emergent literacy skills among Portuguese-speaking children in pre-K, kindergarten, and first grade, including tasks that assess phonological awareness, vocabulary, and concepts about print. This tool is designed to identify children at risk of literacy difficulties early, ensuring there can be timely interventions to prevent future academic challenges. The findings suggest that this tool was suitable for identifying varied literacy-skill levels and had high reliability. Evidence of validity was also provided, as the scores in this tool were correlated with the scores in other standardized tests of emergent literacy, reading and writing, and predicted academic outcomes. As the authors highlight, this tool can be administered in group settings, making it more cost-effective than individual assessments, and is suitable for use by educators and specialists in school contexts.

The study by Morrow et al. (contribution 7) investigates the link between ear health and speech and language outcomes among Indigenous Australian children. Using data from the Longitudinal Study of Indigenous Children, the authors analyze parent-reported ear health at 2–5 years of age and speech/language concerns at ages 5–6. The results suggest that children without parent-reported ear symptoms had lower odds of expressive and especially receptive language concerns a year later. Moreover, parents of male children were more likely to express concern regarding expressive language skills. This study underscores the need for culturally safe, family-centered interventions integrating ear health and speech services to address the needs of this population. Overall, the findings emphasize the importance of community engagement and strengths-based approaches for improving Indigenous children’s health and linguistic outcomes.

The study by Sätilä et al. (contribution 8) explores neuropsychological profiles and challenges among children and adolescents with Borderline Intellectual Functioning (BIF) based on a retrospective analysis of 651 children and adolescents. The results highlight delays in language and motor skill acquisition as common initial concerns and suggest that neuropsychiatric comorbidities were quite prevalent. About ¼ of the participants were later diagnosed with an intellectual disability, and most exhibited persistent deficits in executive functions, reading, arithmetic, and adaptive skills, with these difficulties often worsening over time, especially as academic and life demands increased. This study stresses the importance of early intervention, continuous monitoring, and re-assessment of cognitive and adaptive functions before adulthood to ensure appropriate support for and identification of evolving intellectual disabilities. It also calls for integrated efforts among medical, educational, and social services to improve outcomes for this vulnerable population.

Mustonen et al. (contribution 9) studied the association between screen time and language development in preschool-aged children, considering the influence of mothers’ screen time. This study involved 164 children, aged 2.5 to 4.1 years old, and their mothers. Screen-time data and language assessments revealed that the children’s solo screen use was linked to weaker lexical and general language abilities. Co-viewing with parents showed potential positive associations, though not consistently significant when accounting for age, maternal education, and birth order. Mothers’ screen time correlated with reduced language outcomes among children, likely due to decreased or disrupted parent–child interaction. Importantly, combined extensive screen use for children and mothers had cumulative negative effects. The findings suggest there is a need to limit screen time for both children and parents and promote shared, interactive, and educational content to support language development.

## 3. Conclusions

This Special Issue underscores the dynamic interplay between cognitive and linguistic development, highlighting how these processes relate to each other across diverse contexts and populations. The contributions presented provide novel insights into the mechanisms underlying typical and atypical cognitive and linguistic development, emphasizing critical factors such as executive functions, genetic underpinnings, and environmental influences like screen time and cultural factors. They further suggest that specific differences may emerge, which are linked to the characteristics of the spoken and written language (contribution 1) and individual features such as those related to gender (contributions 1 and 7), age (contributions 3, 6, and 8), and ethnicity (contributions 2 and 7).

More precisely, the findings from the study on children’s interpretations of ambiguous language (contribution 3) suggest that linguistic and cognitive strategies evolve with age, highlighting the importance of tailoring interventions to specific developmental stages. The contributions addressing children with ASD, epilepsy, and borderline intellectual functioning (contributions 4 and 8) emphasize the compounded challenges these conditions pose to cognitive and linguistic development. These studies call for integrative, individualized approaches that address the interplay of multiple factors influencing developmental outcomes. Additionally, the study on Indigenous Australian children (contribution 7) and the assessment of executive function tools for diverse populations (contribution 2) highlights the need for culturally sensitive methods and interventions. These studies advocate for community engagement and the development of accessible, inclusive tools to support diverse linguistic and cognitive needs. Finally, the studies presenting tools for assessing emergent literacy skills (contribution 6) and genetic analyses associated with language impairments (contribution 5) represent promising advances for future research, as they pave the way for the early identification of at-risk children and a deeper understanding of the biological basis of linguistic deficits.

Overall, the studies collectively emphasize the critical need for early intervention, multidisciplinary approaches, and the continuous monitoring of cognitive and linguistic development. By addressing both individual and environmental factors, these efforts can help optimize developmental outcomes and support lifelong learning and adaptation.
